# Comparative analysis of PSMA-RADS Version 2.0 versus bone uptake metastatic probability (BUMP) score for characterising focal bone uptake on [18F]PSMA-1007 PET/CT in prostate cancer – a multicentre evaluation

**DOI:** 10.1007/s00259-026-08154-0

**Published:** 2026-09-01

**Authors:** James Cairns, Salman Arain, Amarnath Challapalli, Amalia Stavropoulos, Tamir Ali, Daniel Ward, John Sage, Georgia Ionescu, Julien M. Y. Willaime, Russell Frood, Amit Bahl, George Petrides, Andrew Scarsbrook

**Affiliations:** 1 Department of Radiology, Leeds Teaching Hospitals NHS Trust, Leeds, UK; 2 Faculty of Medicine and Health, University of Leeds, Leeds, UK; 3 University Hospitals Bristol and Weston NHS Foundation Trust, Bristol, UK; 4 Newcastle Upon Tyne Hospitals NHS Foundation Trust, Newcastle, UK; 5 Mirada Medical Ltd, Oxford, UK

**Keywords:** Prostate cancer, Prostatic cancer, Metastatic, Urology, Diagnostic Techniques, Urological, PSMA-1007

## Abstract

**Purpose:**

Prostate-specific membrane antigen (PSMA) PET/CT is widely used in prostate cancer management, but unspecific bone uptake (UBU) remains a diagnostic challenge particularly with [18F]PSMA-1007. Bone uptake metastatic probability (BUMP) score - a composite imaging-clinical model, was compared with PSMA Reporting and Data System (PSMA-RADS, Version 2) for predicting skeletal metastases in UBU.

**Methods:**

A real-world cohort of [18F]PSMA-1007 PET/CT scans performed at three tertiary cancer centres was retrospectively analysed. Bone lesions with focal PSMA uptake were evaluated. SUV_max_, CT-derived mean Hounsfield unit and androgen deprivation therapy status were recorded to calculate BUMP score for each lesion. Lesional PSMA-RADS scores were assigned by experienced radiologists. Ground truth was established using follow-up imaging, prostate-specific antigen (PSA) kinetics and multidisciplinary consensus. Receiver operating characteristic (ROC) analyses assessed diagnostic performance.

**Results:**

130 patients (median age 70 years) with 412 bone lesions were analysed; 60.9% (*n* = 251) of lesions were metastatic. Median SUV_max_ was higher in malignant than benign lesions: 7.54 (Interquartile range (IQR) 10.3) versus 3.53 (IQR 1.51). BUMP achieved an AUROC of 0.812 (95% CI: 0.636—0.931), the optimal threshold was 0.134 yielding an F1 score of 0.800. PSMA-RADS v2.0 outperformed BUMP with higher sensitivity and specificity (98% and 96%, versus 74% and 80%). In equivocal PSMA-RADS Score 3 lesions (*n* = 44), BUMP showed reduced discrimination (AUROC 0.61, 95% CI 0.457–0.781).

**Conclusion:**

BUMP had good predictive ability for classifying bone metastases but underperformed compared with human-led PSMA-RADS assessment. For equivocal lesions BUMP offered limited added value, highlighting the importance of expert interpretation.

**Supplementary Information:**

The online version contains supplementary material available at https://doi.org/10.1007/s00259-026-08154-0.

## Introduction

Prostate cancer is the second commonest cancer in men and the fourth most common cancer overall worldwide. There were an estimated 1,467,854 new cases diagnosed and 397,430 deaths globally in 2022 [1]. Incidence is projected to increase dramatically with new cases expected to rise from 1.4 million in 2020 to 2.9 million by 2040, driven primarily by ageing populations and improved life expectancy [2]. Accurate staging at diagnosis and biochemical recurrence is critical for guiding treatment decisions, monitoring response and estimating prognosis. The introduction of prostate-specific membrane antigen positron emission tomography/computed tomography (PSMA PET/CT) has significantly improved detection of primary and metastatic disease, offering superior sensitivity and specificity compared with conventional imaging modalities such as bone scintigraphy and CT alone [3]. In addition, PSMA PET/CT has shown the potential to significantly reduce radiation exposure with fewer equivocal results when compared to conventional imaging with CT and bone scintigraphy. Among available radiotracers [18F]PSMA-1007 has gained increasing clinical adoption, demonstrating excellent biodistribution characteristics enabling assessment of the prostate and pelvic region without urinary interference. The tracer demonstrates high tumour-to-background ratio with higher spatial resolution and a longer half-life compared to 68 Ga-labelled PSMA tracers [4].

Despite these advantages, unspecific bone uptake (UBU) on [18F]PSMA-1007 PET/CT is more prevalent with this radiopharmaceutical and represents a persistent and clinically significant diagnostic challenge. UBU is defined as focal bone radiotracer uptake without definitive morphological correlate on CT, or uptake patterns insufficient to confidently distinguish between benign processes and true skeletal metastases. The clinical prevalence of UBU on PSMA PET/CT is substantial, with studies reporting rates of between 19.8–36.7% of patients having at least one indeterminate lesion [5, 6]. Clinical consequences of this uncertainty are multifaceted. Equivocal findings frequently necessitate additional imaging studies, prolonged follow-up protocols and can result in delayed treatment initiation, increased healthcare costs and patient anxiety. More critically, false-positive misinterpretation of UBU can lead to inappropriate upstaging resulting in unnecessary systemic therapy or exclusion from potentially curative treatments. False-negative interpretations may delay necessary interventions for truly metastatic disease. In one study, PSMA PET/CT findings were shown to influence treatment decisions in up to 28% of cases, emphasising the critical importance of accurate lesion characterisation [7].

To address interpretation variability and standardise reporting, several interpretation systems have been developed, notably the PSMA Reporting and Data System (PSMA-RADS) Version 2.0 [8, 9]. PSMA-RADS employs a 5-point ordinal scale ranging from certainly benign (PSMA-RADS 1) to highly suspicious for malignancy (PSMA-RADS 5), with indeterminate findings described as PSMA-RADS 3. The use of PSMA-RADS v2.0 has demonstrated excellent accuracy of 97.4%, 94.7% and 95.7% in new diagnoses, follow-up and biochemical recurrence cases respectively [9]. While PSMA-RADS demonstrates excellent interobserver agreement [10], indeterminate lesions classified as RADS-3 continue to present diagnostic uncertainty.

To address the limitations of visual assessment and structured reporting systems, quantitative methods incorporating imaging and clinical data have been explored as promising solutions. The bone uptake metastatic probability (BUMP) score represents a composite model designed to distinguish UBU from true bone metastases on [18F]PSMA-1007 PET/CT [5]. BUMP score integrates PET-derived maximum standardised uptake values (SUV_max_), CT based mean Hounsfield units (HU_mean_) as a measure of lesion density and androgen deprivation therapy (ADT) status at the time of imaging to generate a composite prediction score for malignancy. BUMP score demonstrated robust diagnostic performance in internal validation, achieving an area under the receiver operating characteristic curve (AUROC) of 0.871 (*n* = 448 bone uptake, 42% metastatic), with external validation AUROC of 0.925 (*n* = 89 bone uptake, 51% metastatic) [5]. The continuous nature enables threshold-based decision making tailored to the specific clinical priority and patient-specific factors.

Despite initial promise, external validation remains limited. There is a paucity of evidence demonstrating whether BUMP score can effectively reduce diagnostic ambiguity specifically in equivocal lesions which represents the clinical scenario where quantitative tools could provide maximum clinical benefit with direct impact on treatment decision making. In addition, there is limited evidence comparing the performance of BUMP score against structured reporting systems such as PSMA-RADS.

Therefore, the present study aimed to further externally validate diagnostic performance of BUMP score for prediction of skeletal metastases in bone lesions detected on PSMA PET/CT across a multi-centre patient cohort. The purpose was also to compare the performance of BUMP score with PSMA-RADS version 2.0 and assess the utility of BUMP score for further stratifying indeterminate PSMA-RADS lesions.

## Methods

### Study design and patient cohort

This was a retrospective, multi-centre study including patients with prostate cancer undergoing [18F]PSMA-1007 PET/CT scans as part of routine clinical care at three tertiary cancer centres in the UK between October 2021 and March 2023.

PET/CT imaging was performed across the three centres using GE Healthcare Discovery 690 and 710 PET/CT scanners. Standardised radiotracer injection protocols and image acquisition parameters followed European Association of Nuclear Medicine (EANM) guidelines [11]. Proprietary ordered subset expectation maximisation (OSEM) time-of-flight reconstructions were performed at each site by default (Vue Point FX, VPFX, GE Healthcare) and used for imaging evaluation.

### Ethical approval

This study was performed in line with the principles of the Declaration of Helsinki and was considered by each participating institution as an observational service evaluation project with no impact on patient care. Formal ethics committee approval and informed patient consent was waived.

### Imaging analysis and lesion selection

[18F]PSMA-1007 PET/CT scans were retrospectively reviewed to identify bone lesions demonstrating focal PSMA uptake. Lesions were included if they exhibited discrete uptake above local background activity and were not definitively physiological uptake. Lesions were segmented by a panel of experienced nuclear medicine and radiology reporters, all of whom were blinded to the adjudicated clinical outcomes. Segmentation was undertaken using a paired-review process in which one reporter delineated the lesion on standard PET/CT visualisation software (XD; Mirada Medical, Oxford, UK) and a second reporter checked and confirmed the contour. Any discrepancies were resolved by consensus. Image review and lesion delineation were undertaken by appropriately credentialed radionuclide radiologists, including dual-certified radiologists/nuclear medicine physicians, with experience in PSMA PET/CT reporting ranging from 1 to more than 5 years. A detailed description of the image-review and annotation procedure and a blank copy of the patient- and lesion-level data-collection form are provided as Supplementary Materials 1 and 2, respectively. For each lesion, the following quantitative metrics were extracted:

- SUV_max_ (Maximum standardised uptake value) values from VPFX PET reconstruction, recorded as the highest pixel value within a manually drawn volume of interest (VOI) encompassing the lesion.
- HU_mean_ (Mean Hounsfield unit) from the corresponding CT component of the PET/CT images.

At patient level, androgen-deprivation therapy (ADT) status at imaging, previous definitive treatment, ISUP grade group and, for patients with biochemical recurrence, PSA doubling time (PSA-DT) at imaging were recorded where available. Patients with biochemical recurrence were classified according to European Association of Urology criteria when the necessary variables were available. Following radical prostatectomy, low-risk biochemical recurrence required a PSA-DT of > 12 months and pathological ISUP grade group* <* 4, whereas high-risk biochemical recurrence was defined by a PSA-DT of ≤ 12 months or pathological ISUP grade group 4-5. Following definitive radiotherapy, low-risk biochemical recurrence required an interval to biochemical failure of > 18 months and biopsy ISUP grade group* <* 4, whereas high-risk biochemical recurrence was defined by an interval of ≤ 18 months or biopsy ISUP grade group 4-5. Patients with insufficient information were reported as not classifiable [12, 13]. The BUMP score for each lesion was calculated using the combined values of SUV_max_, HU_mean_ and ADT status (at the time of scanning), based on the published formula by Bauckneht et al. (https://scorecalc.shinyapps.io/BUMP/) [5].

PSMA-RADS v2.0 categories (1 to 5) were assigned to each lesion by a single experienced reporter to assess agreement against the BUMP score. PSMA-RADS v2.0 scoring was agreed in consensus by a second unblinded reader. PSMA-RADS 3 represented equivocal lesions.

To facilitate direct comparison between BUMP score and PSMA-RADS v2.0 performance lesions labelled PSMA-RADS 3 (equivocal) were removed from confusion matrices and diagnostic performance metric analysis. Sensitivity analysis evaluating the impact of including lesions labelled PSMA-RADS 3 and treating them as benign or malignant was performed. In addition, a patient-level sensitivity analysis was performed to assess the influence of unequal numbers of lesions contributed by individual patients.

### Reference standard for ground truth

Ground truth classification of each lesion (benign, indeterminate or malignant) was established using a composite reference standard which involved review of electronic patient records of individual patients to obtain relevant clinical outcome data. This incorporated:

- Follow-up imaging: Progression (increase in size/number or new lesions) or stability/resolution on subsequent imaging.
- Prostate-specific antigen (PSA) kinetics: PSA response to treatment or rising PSA without other clear cause.
- Multi-disciplinary clinical consensus: decision by the clinical or study team after reviewing all available clinical, biochemical and imaging data.

Lesions lacking adequate follow-up data were deemed indeterminate and excluded from analysis.

### Statistical analysis

All statistical analysis and graphical representation generation was performed in R (version 4.5.2) and Python (3.14.0). Comparison of continuous lesion characteristic variables was performed using linear mixed effects statistical testing with a significance level of *p <* 0.05. Linear mixed effects models were used to account for intra-patient clustering arising from the inclusion of multiple lesions from the same patient.

BUMP and PSMA-RADS v2.0 discrimination for identifying malignant lesions were assessed using receiver operating characteristic (ROC) analysis. ROC curves were constructed and the area under the ROC curve (AUROC) computed. To facilitate comparison of BUMP vs PSMA-RADS v2.0 while accounting for within-patient correlation of multiple lesions, patient-level bootstrap resampling (*n* = 1000 iterations) was performed. Patients were sampled with replacement, including all their lesions, preserving clustering structure. AUROCs and their differences were calculated for each iteration. The 95% confidence intervals were derived from bootstrap percentiles and statistical significance (*p <* 0.05) was assessed via two-tailed bootstrap testing.

The Liu criterion was utilised to derive the optimum local threshold for BUMP score in characterising benign/malignant lesions [14]. This technique matches the methodology used in development and validation of BUMP score [5].

Confusion matrices and diagnostic performance statistics were generated at two key thresholds of BUMP score for comparison:

- Literature derived cut-off (0.25): Obtained from Bauckneht et al.’s publication [5].
- Locally derived optimum cut-off: Determined by the Liu criterion [14] in this new cohort.

Confusion matrices and diagnostic performance statistics were also generated for PSMA-RADS v2.0 scoring.

## Results

**Fig. 1:**
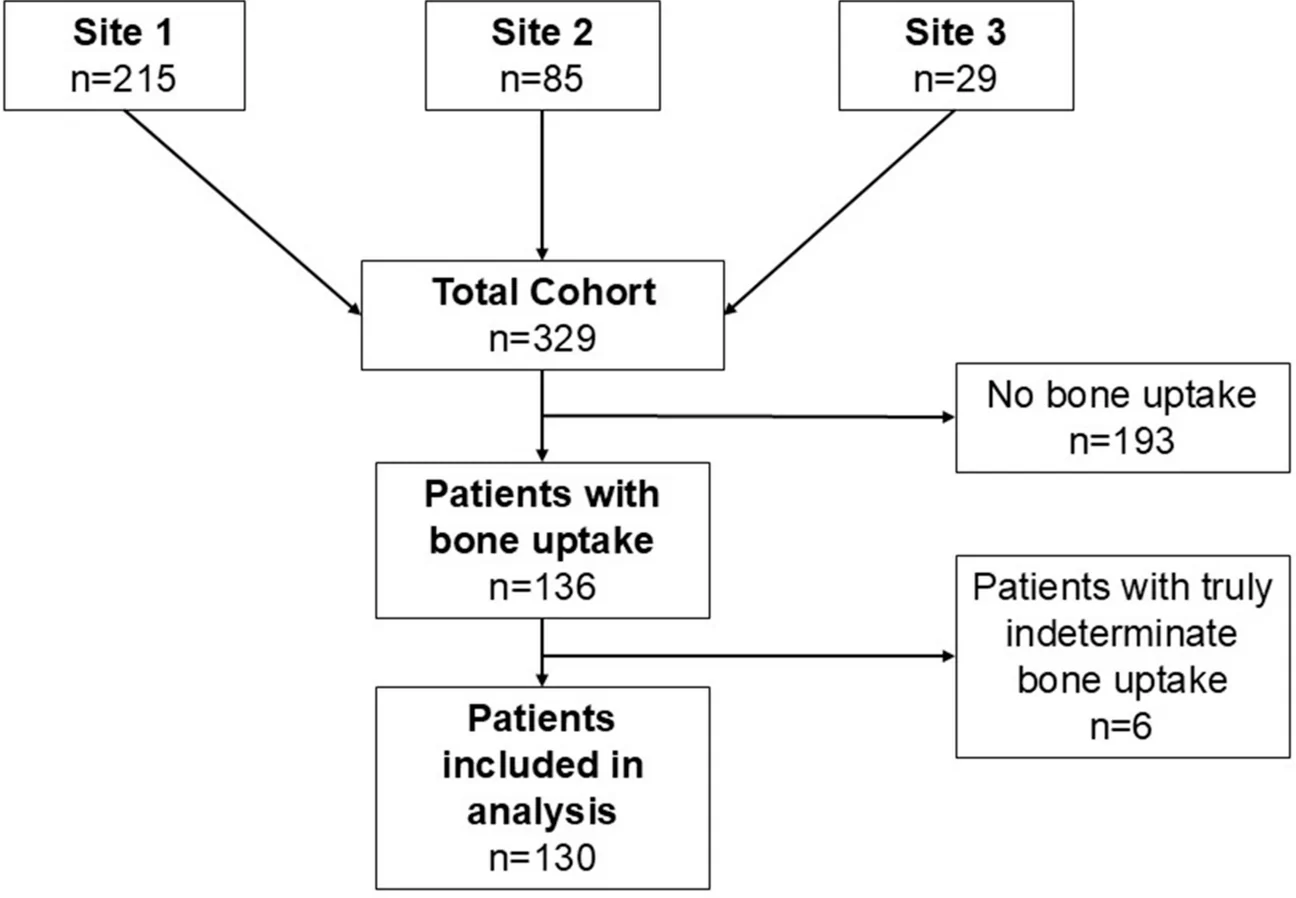
Patient Inclusion Flowchart. Lesions were classified as indeterminate when the available follow-up imaging, PSA kinetics and multidisciplinary assessment were insufficient to assign benign or malignant ground truth with adequate confidence. No prespecified minimum duration of follow-up was imposed

Patient and lesion characteristics are displayed in Tables 1, 2 and 3. A total of 130 patients (median age 70 years) with 412 bone lesions were included in ROC analysis (Fig. 1). Of these, 251 lesions (60.9%) were confirmed to be malignant prostate cancer metastases by multidisciplinary consensus. 161 lesions (39.1%) were deemed to represent benign lesions. The median follow-up length was 22.8 months (IQR 18.8, Range 1.3–130.8).

**Table 1:** Patient characteristics stratified by bone lesion classification (benign or malignant)

	All	*Benign*	*Malignant*
**Patients**			
n	**130**	79 (60.8%)	51 (39.2%)
**Median patient age in years (IQR, range)**			
	**70 (9, 49–84)**	70 (10, 49–81)	70 (9, 51–84)
**Median lesions per patient (IQR, range)**			
n	**2 (2, 1–63)**	1 (1, 1–7)	2 (3.5, 1–63)
**PET-CT Indication**			
Restaging (BCR)	**111 (85.4%)**	68 (61.3%)	43 (38.7%)
Restaging (CRPC)	**3 (2.3%)**	0 (0.0%)	3 (100.0%)
Staging	**8 (6.2%)**	6 (75.0%)	2 (25.0%)
Follow-up	**2 (1.5%)**	1 (50.0%)	1 (50.0%)
Surveillance of indeterminate findings	**4 (3.1%)**	3 (75.0%)	1 (25.0%)
Missing data	**2 (1.5%)**	1 (50.0%)	1 (50.0%)
**ADT status at time of PET/CT**			
Yes	**19 (14.6%)**	9 (47.4%)	10 (52.6%)
No	**111 (85.4%)**	70 (63.1%)	41 (36.9%)
**Prostate-Specific Antigen (PSA) Kinetics (IQR, range)**			
Median PSA at diagnosis	**9.52 (8.82, 3.1–180)**	10.33 (8.55, 3.1–180)	8.84 (9.22, 4.3–125)
Median PSA closest to PET/CT	**1.40 (2.63, 0.2–100)**	1.0 (1.76, 0.2–100)	2.16 (3.18, 0.25–25)
Median PSA at last follow-up	**0.1 (0.94, 0.006-** **224.0)**	0.1 (1.11, 0.007–21.8)	0.1 (0.17, 0.006- 224.0)
**Initial T Stage**			
T1a	**0 (0.0%)**	0 (0.0%)	0 (0.0%)
T1b	**1 (0.8%)**	0 (0.0%)	1 (100.0%)
T1c	**2 (1.5%)**	1 (50.0%)	1 (50.0%)
T2a	**12 (9.2%)**	8 (66.7%)	4 (33.3%)
T2b	**1 (0.8%)**	1 (100.0%)	0 (0.0%)
T2c	**7 (5.4%)**	6 (85.7%)	1 (14.3%)
T3a	**46 (35.4%)**	25 (54.3%)	21 (45.7%)
T3b	**34 (26.2%)**	21 (61.8%)	13 (38.2%)
T4	**0 (0.0%)**	0 (0.0%)	0 (0.0%)
Missing data	**27 (20.8%)**	17 (63.0%)	10 (37.0%)
**Initial N Stage**			
N0	**74 (56.9%)**	45 (60.8%)	29 (39.2%)
N1	**23 (17.7%)**	13 (56.5%)	10 (43.5%)
Nx	**6 (4.6%)**	4 (66.7%)	2 (33.3%)
Missing data	**27 (20.8%)**	17 (63.0%)	10 (37.0%)
**Initial M Stage**			
M0	**98 (75.4%)**	60 (61.2%)	38 (38.8%)
M1a (non-regional nodes)	**2 (1.5%)**	1 (50.0%)	1 (50.0%)
M1b (bone)	**3 (2.3%)**	1 (33.3%)	2 (66.7%)
Missing data	**27 (20.8%)**	17 (63.0%)	10 (37.0%)
**ISUP Grade**			
ISUP 1	**7 (5.4%)**	6 (85.7%)	1 (14.3%)
ISUP 2	**43 (33.1%)**	26 (60.5%)	17 (39.5%)
ISUP 3	**34 (26.2%)**	24 (70.6%)	10 (29.4%)
ISUP 4	**18 (13.8%)**	11 (61.1%)	7 (38.9%)
ISUP 5	**20 (15.4%)**	8 (40.0%)	12 (60.0%)
Missing data	**8 (6.2%)**	4 (50.0%)	4 (50.0%)
**Treatment Modality Before PET/CT**			
External beam radiotherapy	**41 (31.5%)**	18 (43.9%)	23 (56.1%)
Radical prostatectomy	**84 (64.6%)**	58 (69.0%)	26 (31.0%)
Post-prostatectomy EBRT	**32 (24.6%)**	19 (59.4%)	13 (40.6%)
ADT previous/ongoing	**57 (43.8%)**	28 (49.1%)	29 (50.9%)
Brachytherapy	**8 (6.2%)**	4 (50.0%)	4 (50.0%)
Chemotherapy	**6 (4.6%)**	3 (50.0%)	3 (50.0%)
Missing data	**10 (7.7%)**	6 (60.0%)	4 (40.0%)
**Treatment Modality After PET/CT**			
Chemotherapy/AR-targeted agents	**55 (42.3%)**	20 (36.4%)	35 (63.6%)
Androgen deprivation therapy	**69 (53.1%)**	38 (55.1%)	31 (44.9%)
Targeted radiotherapy	**5 (3.8%)**	2 (40.0%)	3 (60.0%)
Salvage brachytherapy	**2 (1.5%)**	1 (50.0%)	1 (50.0%)
Radiotherapy to prostate bed ± nodes	**14 (10.8%)**	14 (100.0%)	0 (0.0%)
Radiotherapy to lymph nodes	**11 (8.5%)**	10 (90.9%)	1 (9.1%)
Salvage RT to prostatectomy bed	**9 (6.9%)**	7 (77.8%)	2 (22.2%)
Stereotactic RT to bone	**12 (9.2%)**	1 (8.3%)	11 (91.7%)
Radioligand therapy	**1 (0.8%)**	0 (0.0%)	1 (100.0%)
Radiotherapy to prostate	**1 (0.8%)**	0 (0.0%)	1 (100.0%)
No treatment	**19 (14.6%)**	18 (94.7%)	1 (5.3%)
Missing data	**12 (9.2%)**	10 (83.3%)	2 (16.7%)
**Median follow-up duration (months) (IQR, Range)**			
	**22.8 (18.8, 1.3–130.8)**	21.7 (19.5, 1.8–48.0)	24.0 (18.5, 1.3–130.8)
**Subsequent Imaging**			
Yes - MRI	**24 (18.5%)**	16 (66.7%)	8 (33.3%)
Yes - CT	**2 (1.5%)**	1 (50.0%)	1 (50.0%)
Yes - MRI and PSMA PET-CT	**6 (4.6%)**	6 (100.0%)	0 (0.0%)
Yes - follow-up PSMA PET-CT	**17 (13.1%)**	12 (70.6%)	5 (29.4%)
Yes - Bone scan	**4 (3.1%)**	2 (50.0%)	2 (50.0%)
No	**71 (54.6%)**	37 (52.1%)	34 (47.9%)
Missing data	**6 (4.6%)**	5 (83.3%)	1 (16.7%)

Patients were classified as ‘malignant’ if they demonstrated any malignant bone lesions on PSMA PET/CT defined by multidisciplinary consensus. Patients with only benign bone lesions were classified as ‘benign’. Some patients received more than one treatment modality before or after PSMA PET-CT scanning. *BCR*, biochemical recurrence, *CRPC*, castration resistant prostate cancer, *EBRT*, external beam radiotherapy, *RT*, radiotherapy, *ISUP*, international society of urological pathology, *ADT*, androgen deprivation therapy, *TNM*, tumour-node-metastasis, *IQR*, interquartile range, *MRI*, magnetic resonance imaging, *AR-targeted*, androgen receptor-targeted

**Table 2:** Biochemical-recurrence subgroup characteristics

	All	Benign	Malignant	*P*-value
**Patients with biochemical recurrence, n**				
	111	68	43	N/A
**PSA-Doubling Time Available, n (%)**				
	57 (51.4%)	37 (54.4%)	20 (46.5%)	0.442†
**Median PSA-Doubling Time, months (IQR;range)**				
	6.0 (4.3–11.0;0.9–194.0)	6.9 (4.4–11.9; 1.2–29.0)	5.1 (3.0–6.2; 0.9–194.0)	0.047‡
**EAU risk**				
High-risk BCR	62 (55.9%)	38 (55.9%)	24 (55.8%)	0.609†
Low-risk BCR	21 (18.9%)	11 (16.2%)	10 (23.3%)	
BCR risk not classifiable	28 (25.2%)	19 (27.9%)	9 (20.9%)	

PSA-DT and EAU risk percentages use the biochemical-recurrence subgroup denominators: all, *n* = 111; benign, *n* = 68; malignant, *n* = 43. EAU percentages include patients who were not classifiable. PSA-DT, PSA doubling time; BCR, biochemical recurrence; EAU, European Association of Urology. †Two-sided Fisher exact test; the EAU comparison included only classifiable patients. ‡Two-sided Mann–Whitney U test

**Table 3:** Lesion characteristics stratified by lesion classification (benign or malignant)

	All	Benign	Malignant	*P*-value
**Number of Lesions**				
n	**412**	161	251	N/A
**Anatomical Location of Lesion**				
Pelvic	**63 (15.3%)**	28 (44.4%)	35 (55.6%)	N/A
Vertebrae	**113 (27.4%)**	35 (31.0%)	78 (69.0%)	
Rib	**174 (42.2%)**	81 (46.6%)	93 (53.4%)	
Sacrococcyx	**22 (5.3%)**	8 (36.4%)	14 (63.6%)	
Femur	**12 (2.9%)**	4 (33.3%)	8 (66.7%)	
Manubrium/sternum	**6 (1.5%)**	1 (16.7%)	5 (83.3%)	
Clavicular	**2 (0.5%)**	2 (100.0%)	0 (0.0%)	
Skull	**3 (0.7%)**	2 (66.7%)	1 (33.3%)	
Scapula	**17 (4.1%)**	0 (0.0%)	17 (100.0%)	
**Median lesion size in mm (IQR, Range)**				
	**10.0 (9.0, 2–79)**	6.0 (4.0, 2–25)	12.0 (9.0, 3–79)	*p <* 0.001
Missing data	**3 (0.7%)**	3 (100.0%)	0 (0.0%)	
**Median lesion density in Hounsfield units (IQR, Range)**				
	**235.1 (150.2, 16.1–911.9)**	196.4 (114.9, 16.1–558.1)	259.0 (171.8, 45.2–911.9)	*p <* 0.001
**Median lesion SUVmax (IQR, Range)**				
	**4.90 (5.52, 1.55–82.40)**	3.53 (1.51, 1.55–11.95)	7.54 (10.29, 1.66–82.40)	*p <* 0.001
**Lesion ADT Status**				
Yes	**116 (28.2%)**	19 (16.4%)	97 (83.6%)	N/A
No	**296 (71.8%)**	142 (48.0%)	154 (52.0%)	
**Median lesion BUMP Probability (IQR, Range)**				
	**0.165 (0.563, 0.0222–1.00)**	0.076 (0.067, 0.022–0.682)	0.427 (0.784, 0.023–1.00)	*p <* 0.001
**Presence of Sclerosis Within Lesion**				
Yes	**207 (50.2%)**	8 (3.9%)	199 (96.1%)	N/A
No	**181 (43.9%)**	129 (71.3%)	52 (28.7%)	
Missing data	**24 (5.8%)**	24 (100.0%)	0 (0.0%)	
**Typical Pattern of Lesion Distribution**				
Yes	**305 (74.0%)**	70 (23.0%)	235 (77.0%)	N/A
No	**76 (18.4%)**	64 (84.2%)	12 (15.8%)	
Missing data	**31 (7.5%)**	27 (87.1%)	4 (12.9%)	
**Typical Location of Lesion**				
Yes	**322 (78.2%)**	82 (25.5%)	240 (74.5%)	N/A
No	**61 (14.8%)**	54 (88.5%)	7 (11.5%)	
Missing data	**29 (7.0%)**	25 (86.2%)	4 (13.8%)	
**PSMA-RADS v2**				
Reader confidence 1 (benign)	**22 (5.3%)**	22 (100.0%)	0 (0.0%)	N/A
Reader confidence 2 (probably benign)	**107 (26.0%)**	103 (96.3%)	4 (3.7%)	
Reader confidence 3 (equivocal)	**44 (10.7%)**	31 (70.5%)	13 (29.5%)	
Reader confidence 4 (probably PCa)	**29 (7.0%)**	4 (13.8%)	25 (86.2%)	
Reader confidence 5 (definite PCa)	**210 (51.0%)**	1 (0.5%)	209 (99.5%)	

Continuous variables are displayed as median (interquartile range, range) and categorical/discrete variables as counts. *P*-values represent linear mixed effects statistical testing. *IQR* interquartile range, *SUV*_*max*_ maximum standardised uptake value, *ADT* androgen deprivation therapy, *BUMP* bone uptake metastatic probability. ADT status refers to whether the patient was on ADT at the time of the PET/CT scan

Most patients in the study underwent PSMA PET/CT for restaging due to biochemical recurrence (BCR) (*n* = 111, 85.4%). 111 out of 130 patients (85.4%) were not on ADT at the time of scanning. The most common initial TNM staging was T3a N0 M0 and the median initial serum PSA value was 9.52 (8.82, 3.1–180). Among the 111 patients with biochemical recurrence, numeric PSA-DT was available for 57 (51.4%) (Table 2). Median PSA-DT was shorter in patients with malignant than benign bone lesions (5.1 months (IQR 3.0–6.2) versus 6.9 months (IQR 4.4–11.9); Mann–Whitney U, p = 0.047). EAU biochemical-recurrence risk classification [12, 13] was possible in 83/111 patients (74.8%), of whom 62 (74.7%) were classified as high risk and 21 (25.3%) as low risk. Among classifiable patients, high-risk biochemical recurrence was present in 38/49 (77.6%) patients in the benign group and 24/34 (70.6%) in the malignant group, with no significant difference between groups (Fisher exact p = 0.609). Twenty-eight patients could not be classified because one or more required variables were unavailable.

In patients undergoing PSMA PET/CT in this study, radical prostatectomy (*n* = 84, 64.6%) was the commonest prior treatment followed by external beam radiotherapy (*n* = 73, 56.2%). 57 patients had received prior or ongoing ADT (43.8%).

The most frequent anatomical location for PSMA-positive lesions was the ribs (*n* = 174, 42.2%), followed by the vertebrae (*n* = 113, 27.4%) and pelvis (*n* = 63, 15.3%). The median lesion size for the entire cohort was 10.0 mm (IQR 9.0 mm). Malignant lesions were larger (12.0 mm, IQR 9.0 mm) than benign lesions (6.0 mm, IQR 4.0 mm) (*p <* 0.001).

Malignant lesions showed higher radiotracer uptake with a median SUV_max_ of 7.54 (IQR 10.29) compared to benign lesions (median SUV_max_ 3.53 (1.51) (*p <* 0.001). Malignant lesions demonstrated a higher median Hounsfield unit density (median HU 259.0, IQR 171.8) compared to benign lesions (median HU 196.4, IQR 114.9) (*p <* 0.001).

### BUMP Score Performance

**Fig. 2:**
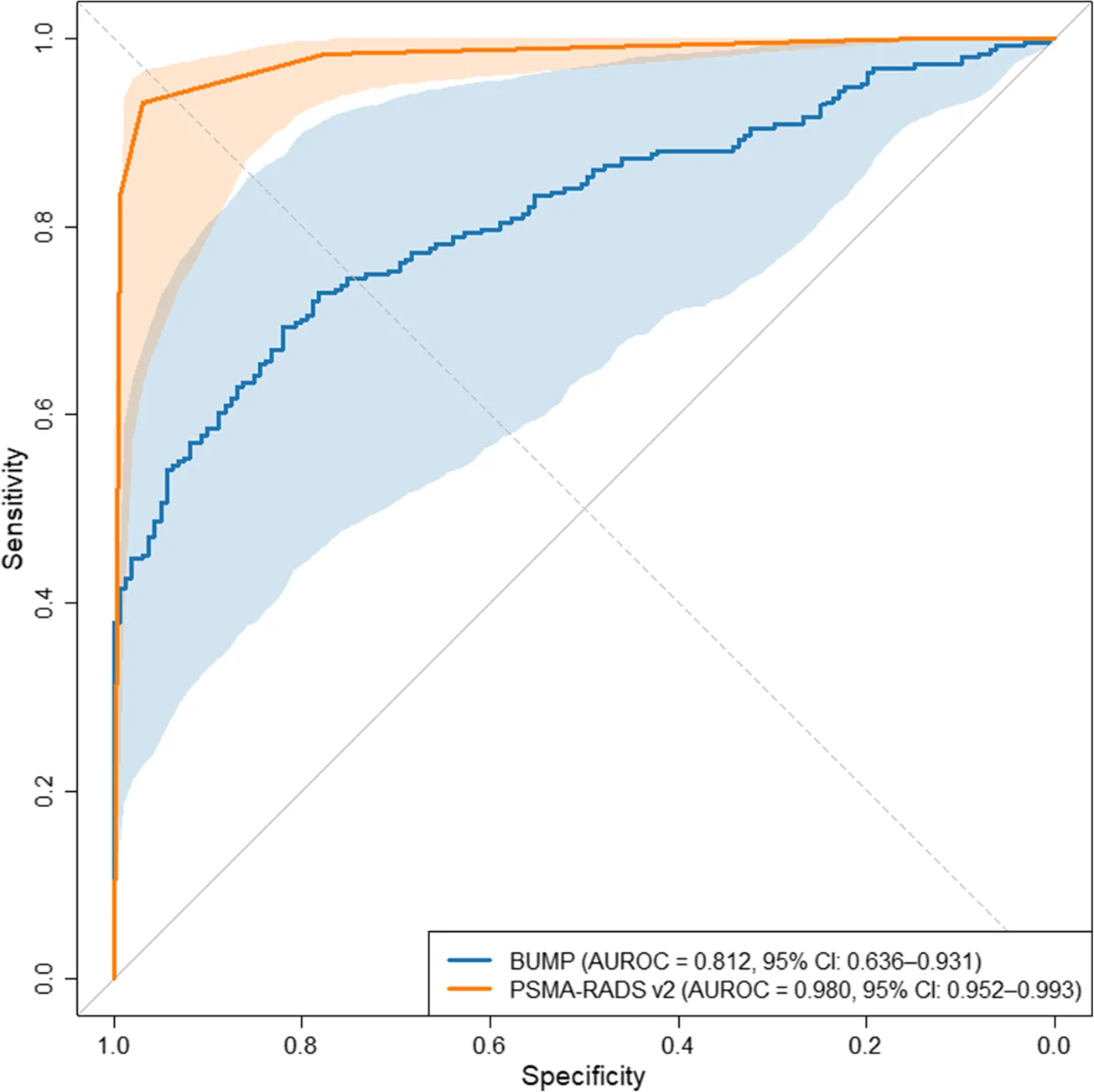
Receiver operating characteristic (ROC) curve demonstrating diagnostic performance of BUMP score and expert PSMA-RADS v2 for characterising benign and malignant bone lesions on PSMA PET/CT. AUROC = Area under the receiver operating characteristic curve

The calculated median BUMP score was higher for malignant lesions (median 0.427, (IQR 0.78)) compared to benign lesions (median 0.08 (IQR 0.07)) (*p <* 0.001). ROC analysis of BUMP score demonstrated an area under the curve (AUROC) of 0.812 (95% CI 0.636–0.931) for discriminating malignant from benign lesions (Fig. 2).

**Fig. 3:**
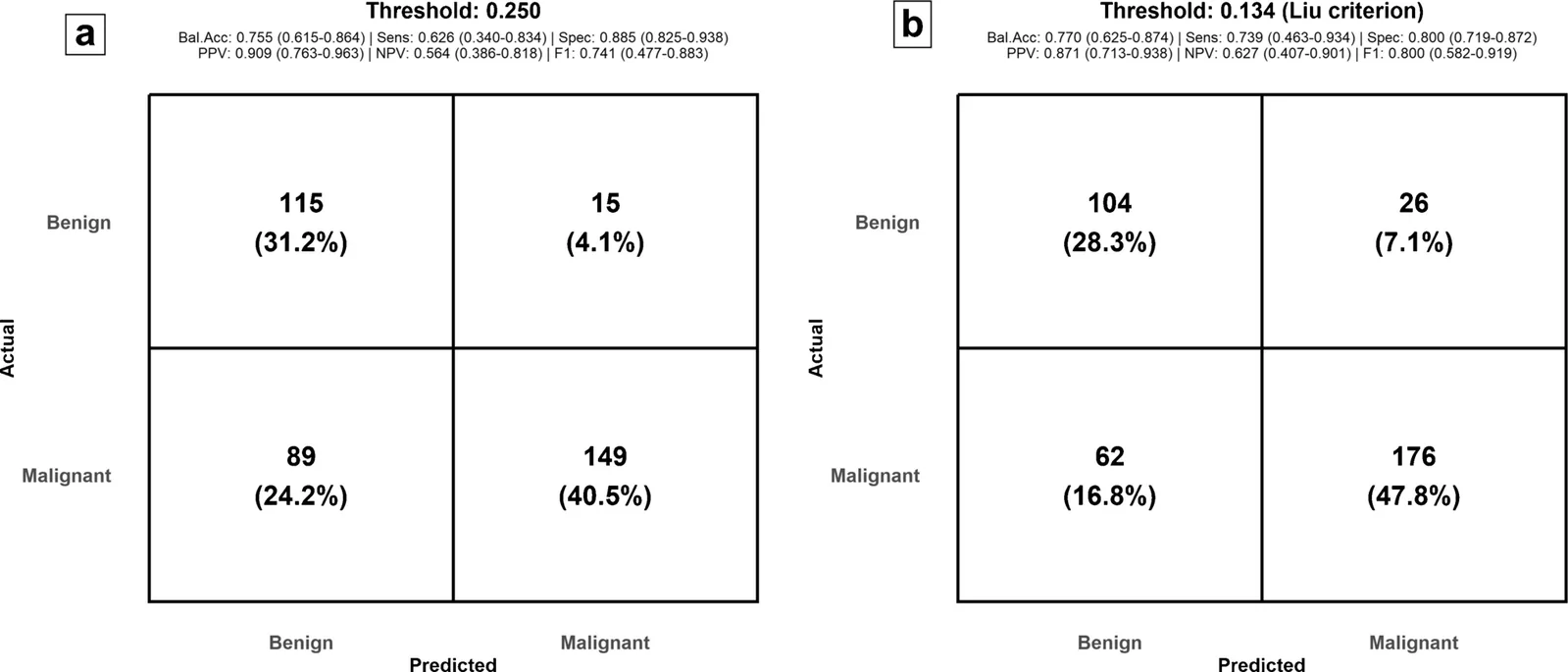
Confusion matrices demonstrating diagnostic performance of BUMP score for characterising benign and malignant bone lesions on PSMA PET/CT. Performance is assessed at both the literature derived probability threshold (**a**) (0.25) and the locally derived probability threshold (**b**) (0.134). Bal.Acc = Balanced Accuracy, Sens = Sensitivity, Spec = Specificity, PPV = positive predictive value, NPV = negative predictive value, F1 = F1 score

The optimal probability threshold using the Liu criterion within the cohort was a threshold of 0.134. At this probability threshold, after exclusion of lesions labelled PSMA-RADS 3 (*n* = 44), BUMP achieved sensitivity of 73.9% (95% CI 46.3–93.4), specificity of 80.0% (95% CI 71.9–87.2), balanced accuracy of 77.0% (95% CI 62.5–87.4) and F1 score 0.800 (95% CI 58.2–91.9). At the literature sourced probability threshold of 0.25, sensitivity decreased (62.6%, 95% CI 34.0–83.4) with improved specificity (88.5%, 95% CI 82.5–93.8), resulting in an F1 score of 0.74 (95% CI 47.7–88.3) (Fig. 3).

### Comparison with expert PSMA-RADS v2.0

In PSMA-RADS v2.0 evaluation, 234 out of 251 (93.2%) malignant lesions were scored as 4: probable prostate cancer or 5: definite prostate cancer. 125 out of 161 (77.6%) benign lesions were scored as benign or probably benign. Score 3 was assigned to 31 benign and 13 malignant lesions.

**Fig. 4:**
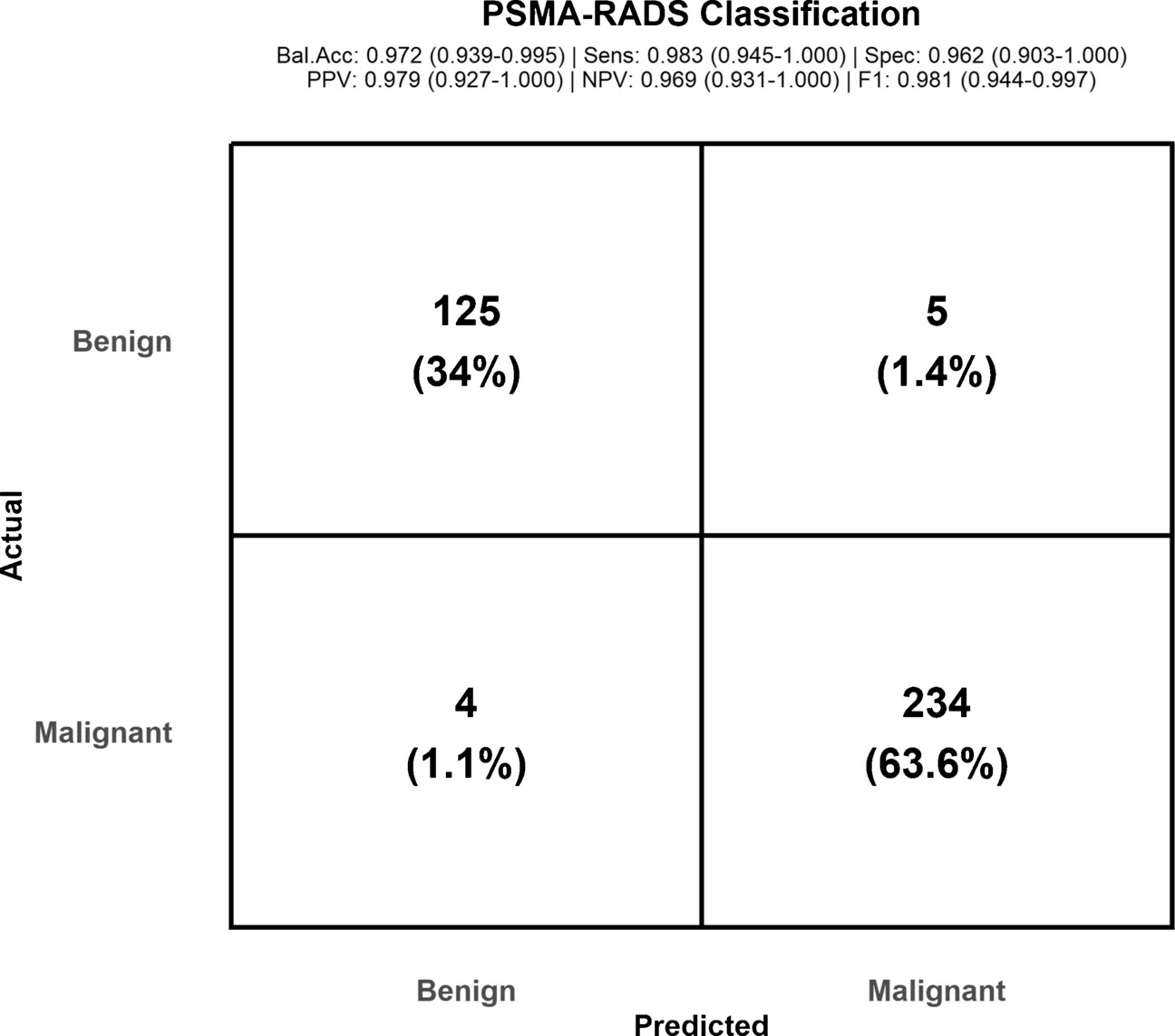
Confusion matrix demonstrating diagnostic performance and sensitivity analysis of human reporting with PSMA-RADS for diagnosing benign vs malignant lesions. PSMA-RADS 3 lesions are excluded. Bal.Acc = Balanced Accuracy, Sens = Sensitivity, Spec = Specificity, PPV = positive predictive value, NPV = negative predictive value, F1 = F1 score

ROC analysis of PSMA-RADS v2.0 scoring demonstrated an AUROC of 0.980 (95% CI 0.952–0.993) (Fig. 2). When PSMA-RADS 1–2 were treated as benign and PSMA-RADS 4–5 as malignant with exclusion of PSMA-RADS 3 lesions (*n* = 44), this resulted in a diagnostic performance with sensitivity 98.3% (95% CI 94.5–100), specificity 96.2% (95% CI 90.3–100), balanced accuracy 97.3% (95% CI 93.9–99.5), F1 score 0.981 (95% CI 94.4–99.7) (Table 3, Fig. 4). PSMA-RADS v2.0 performed better than BUMP score with an AUROC difference of 0.1679 (95% CI: 0.050 to 0.337, *p <* 0.001).

In equivocal PSMA-RADS 3 lesions (*n* = 44) BUMP scoring demonstrated reduced discriminative power with an AUROC of 0.605 (95% CI 0.457–0.781) (Table 3, Supplemental Figure).

### Sensitivity analysis

Sensitivity analysis showed that PSMA-RADS v2.0 retained superior performance to BUMP regardless of how PSMA-RADS 3 lesions were handled. When PSMA-RADS 3 lesions were treated as benign, sensitivity, specificity and balanced accuracy were 93.2% (95% CI 84.2–96.9), 96.9% (95% CI 92.6–100) and 95.1% (95% CI 90.2–97.8), respectively. When PSMA-RADS 3 lesions were treated as malignant, sensitivity increased to 98.4% (95% CI 94.5–100) with reduced specificity of 77.6% (95% CI 67.9–86.1) and balanced accuracy of 88.0% (95% CI 82.8–92.3). By comparison, BUMP showed lower performance across all lesions at both the locally derived threshold of 0.134 and the literature-derived threshold of 0.25, with balanced accuracies of 75.4% (95% CI 62.0–85.4) and 74.4% (95% CI 61.4–85.2), respectively (Table 3).

In the patient-level sensitivity analysis of 130 patients, including 79 without and 51 with skeletal metastatic disease, PSMA-RADS retained superior performance to BUMP. After excluding 24 patients with a maximum PSMA-RADS category of 3, sensitivity was 95.6%, specificity was 95.1% and balanced accuracy was 95.3%. When category 3 was treated as negative, balanced accuracy was 90.3%, compared with 84.7% when treated as positive. For BUMP, balanced accuracy was 75.0% at the 0.134 threshold and 72.2% at the 0.250 threshold. Patient-level AUROC was 0.966 for PSMA-RADS after exclusion of indeterminate patients and 0.840 for BUMP (Table 4).

**Table 4:** Sensitivity analysis demonstrating the impact of including lesions labelled PSMA-RADS 3 on diagnostic performance of BUMP and PSMA-RADS v2. The diagnostic performance of BUMP score in the context of analysing PSMA-RADS 3 lesions only is included. PPV = positive predictive value, NPV = negative predictive value

		Balanced accuracy	Sensitivity	Specificity	PPV	NPV	F1 score
PSMA-RADS v2	**All lesions (*n* = 412)** PSMA-RADS 3 = Benign (1–3 vs 4–5)	0.951 (0.902–0.978)	0.932 (0.842–0.969)	0.969 (0.926–1.000)	0.979 (0.932–1.000)	0.902 (0.841–0.951)	0.955 (0.894–0.980)
	**All lesions (*n* = 412)** PSMA-RADS 3 = Malignant (1–2 vs 3–5)	0.880 (0.828–0.923)	0.984 (0.945–1.000)	0.776 (0.679–0.861)	0.873 (0.732–0.936)	0.969 (0.928–1.000)	0.925 (0.828–0.962)
BUMP Score	**All lesions (*n* = 412)** Threshold 0.134	0.754 (0.620–0.854)	0.725 (0.475–0.911)	0.783 (0.710–0.853)	0.839 (0.681–0.918)	0.646 (0.443–0.871)	0.778 (0.580–0.895)
	**All lesions (*n* = 412)** Threshold 0.250	0.744 (0.614–0.852)	0.606 (0.350–0.811)	0.882 (0.820–0.935)	0.889 (0.747–0.954)	0.589 (0.420–0.793)	0.720 (0.490–0.862)
	**PSMA-RADS 3 lesions only (*n* = 44**) Threshold 0.134	0.586 (0.419–0.730)	0.462 (0.167–0.727)	0.710 (0.552–0.833)	0.400 (0.118–0.667)	0.759 (0.565–0.903)	0.429 (0.160–0.647)
	**PSMA-RADS 3 lesions only (*n* = 44)** Threshold 0.250	0.551 (0.425–0.680)	0.231 (0.000–0.462)	0.871 (0.759–0.968)	0.429 (0.000–0.833)	0.730 (0.545–0.880)	0.300 (0.105–0.545)

**Fig. 5:**
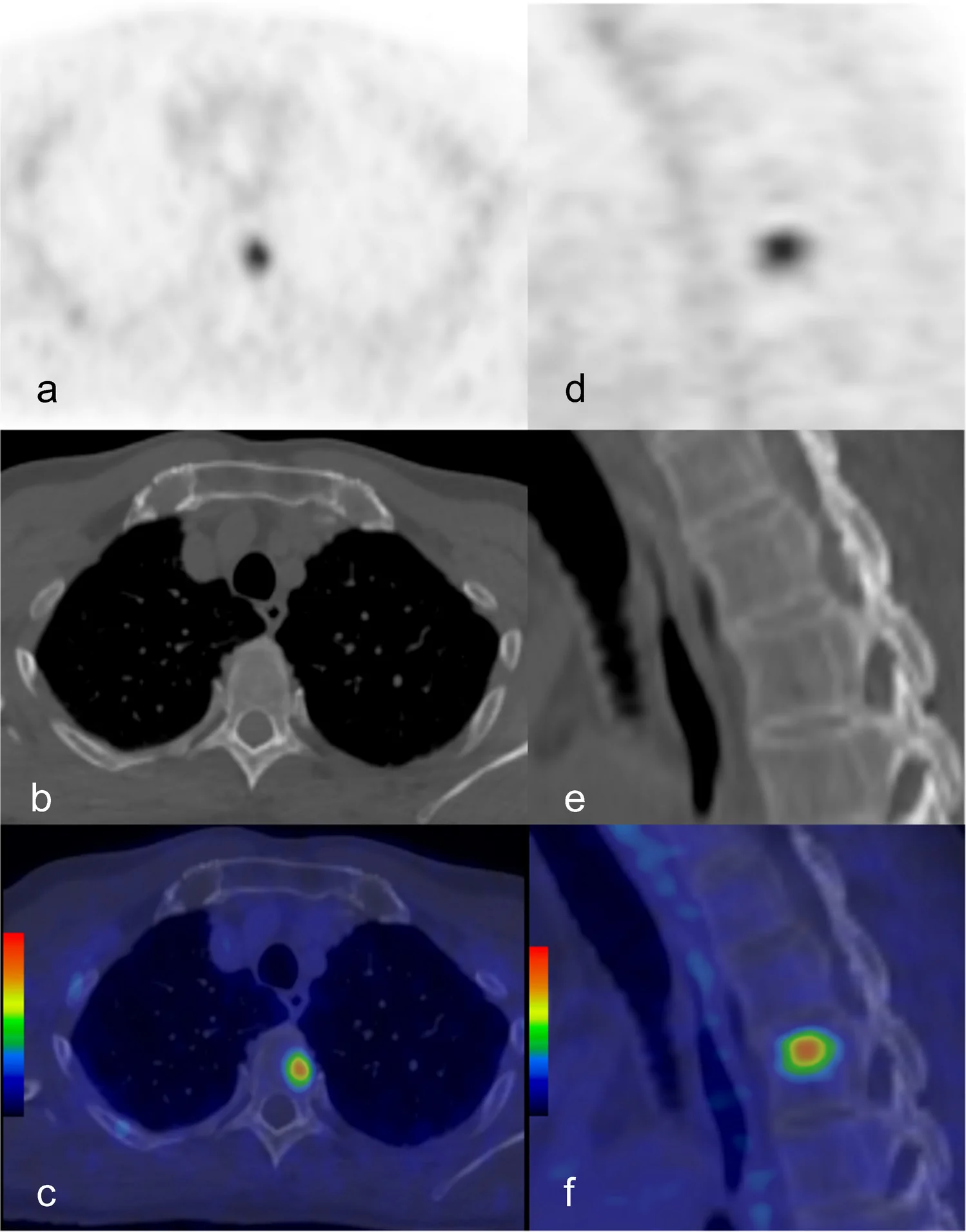
True positive PSMA-RADS/False negative on BUMP. Composite panel of axial and coronal PET (**a**, **d**), low-dose bone window CT (**b**, **e**) and fused PET-CT (**c**, **f**) showing focal uptake in the left side of the T4 vertebral body with subtle correlative lucency (15 mm, SUVmax 5.5) in a 69 yr old patient undergoing restaging PSMA PET-CT for suspected recurrence (PSA 2.12) 3 months following prior prostatectomy for a resection-margin negative T3a N0 M0 ISUP Grade 4 prostate cancer who was not on ADT at the time of imaging. Because of the correlative CT and PET abnormalities this was evaluated as PSMA-RADS 5. BUMP score was calculated at 0.1 (likely benign). Ground truth was determined by adjudicated multidisciplinary consensus as no follow-up imaging was indicated in this case

**Fig. 6:**
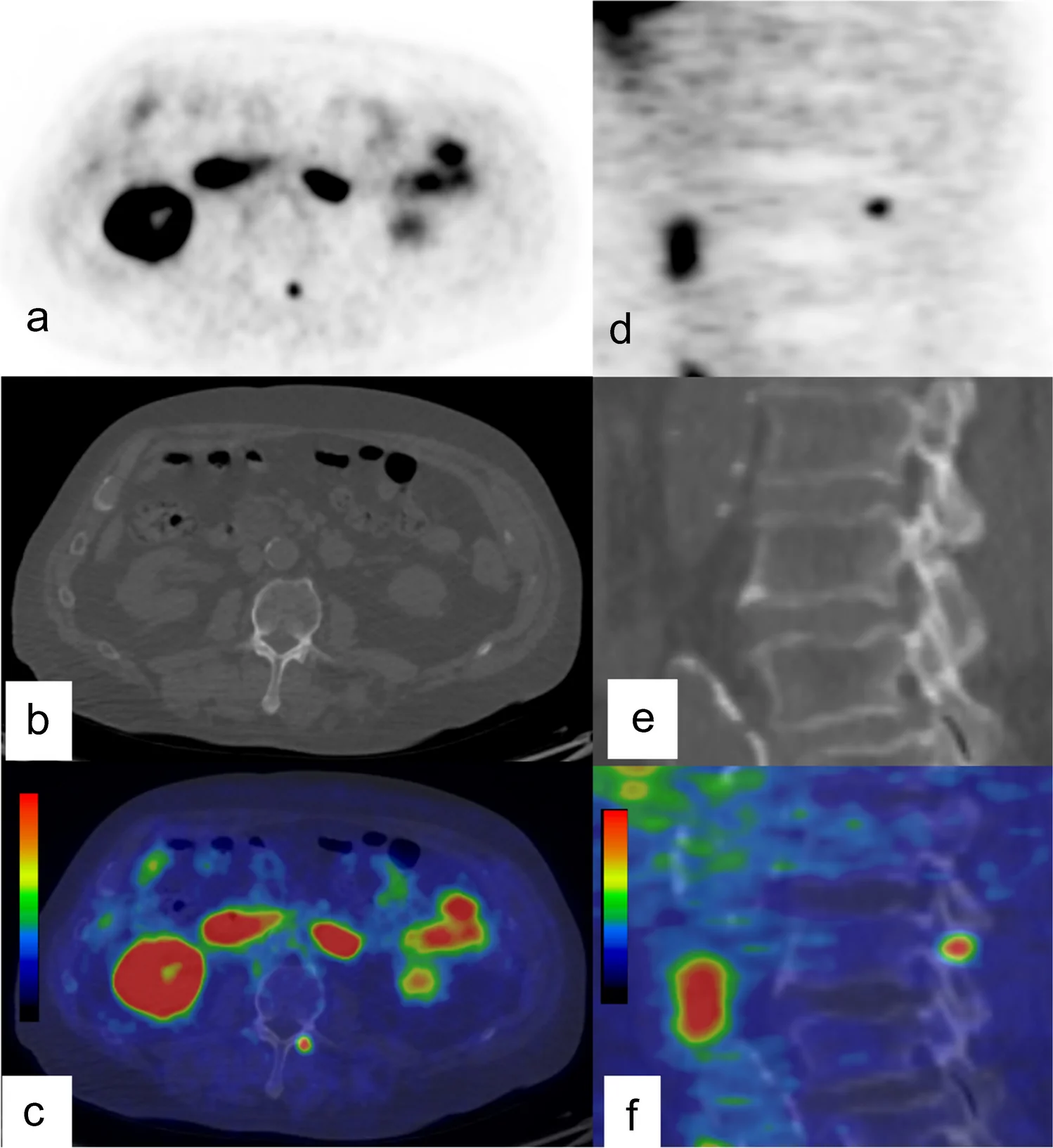
True negative PSMA-RADS/False positive on BUMP. Composite panel of axial and coronal PET (**a**, **d**), low-dose bone window CT (**b**, **e**) and fused PET-CT (**c**, **f**) showing focal uptake in the left sided facet joint of L3 (10 mm, SUVmax 7.6) in an 84 yr old patient undergoing restaging PSMA PET-CT for suspected recurrence (PSA 10.93, PSA doubling time 6.2 months) 4 yrs following starting ADT for biochemical recurrence 6 yrs post radical radiotherapy for a T3a N0 M0 ISUP Grade 5 prostate cancer who was on ADT at the time of imaging. Because of the anatomical location on CT the PET abnormality was evaluated as PSMA-RADS 2 (likely benign). BUMP score was calculated at 0.61 (likely malignant). Ground truth was determined by adjudicated multidisciplinary consensus as no follow-up imaging was indicated in this case

In a few patients there was discrepancy between PSMA-RADS and BUMP classification of osseous lesions. Two cases are illustrated to demonstrate the superior utility of human-directed PSMA-RADS scoring: a true positive vertebral metastasis correctly predicted by PSMA-RADS but classified as benign on BUMP score (Fig. 5); and a focal area of bone activity related to benign facet joint arthropathy which was correctly classified by PSMA-RADS but falsely positive for a metastasis on BUMP score (Fig. 6).

## Discussion

In this multicentre retrospective study, the diagnostic performance of the Bone Uptake Metastatic Probability (BUMP) score with structured reporting using PSMA-RADS Version 2.0 for characterising bone uptake on PSMA-1007 PET/CT scans was compared. The findings demonstrate that although the BUMP score achieved good overall diagnostic performance (AUROC 0.812, 95% CI: 0.636–0.931), it was outperformed by structured expert reporting incorporating PSMA-RADS. The patient-level sensitivity analysis was consistent with the primary lesion-level findings, with PSMA-RADS retaining superior diagnostic performance to BUMP. This suggests that the principal conclusion was not driven solely by greater weighting of patients contributing multiple lesions.

While structured PSMA-RADS assessment demonstrated superior diagnostic accuracy, the reproducibility and objective nature of BUMP score offers potential utility as a decision support adjunct for bone lesions, particularly in high-volume centres or to assist reporters with limited subspecialist expertise. The BUMP score was developed and validated by Bauckneht et al. who reported AUROC values of 0.87 and 0.92 in internal and external validation respectively [5]. In the current multicentre dataset, the AUROC value of 0.812 represents a modest reduction in performance, likely reflecting broader real-world heterogeneity. In addition, the optimum probability threshold in our centres differed. This is a common challenge when generalising quantitative models across different institutions and highlights the importance of local threshold assessment prior to clinical deployment. A benefit of the BUMP score is that parameters are relatively simple to obtain through delineation with a volume of interest and through reviewing the patient record to determine ADT status. More complex radiomic or artificial intelligence analysis of lesions with contextual awareness may better emulate human interpretative reasoning and improve predictive performance [15, 16]. This is supported by a recent multicentre externally validated radiomics-based machine-learning model for [18F]PSMA-1007 bone uptake classification, which demonstrated robust external test performance and significantly improved discrimination compared with SUVmax alone [17].

The lower discriminative power of BUMP scoring in the context of equivocal (PSMA-RADS 3) lesions (AUROC 0.605, 95% CI 0.457–0.781) highlights limitations of quantitative metrics when clinical and anatomical context is not incorporated. Equivocal lesions may demonstrate intermediate SUV_max_ values and ambiguous CT density characteristics. Expert interpretation can integrate lesion distribution, morphology and uptake with overall disease burden and clinical pre-test probability, features that are not represented in BUMP. A systematic review found that ribs were the commonest site of unspecific bone uptake across PSMA tracers and that isolated rib uptake was usually non-metastatic. However, unspecific uptake also occurs in the spine and pelvic girdle, particularly with [18F]PSMA-1007. Lesion location should therefore inform, rather than determine, metastatic probability and should be considered alongside uptake intensity, CT morphology and the overall clinical context [18, 19]. This interpretation is supported by a recent study comparing MDT assessment with PROMISE criteria for indeterminate bone uptake on staging PSMA PET, which demonstrated substantial agreement overall but found that long-term follow-up favoured MDT interpretation in all discordant cases [20]. That study also reported that MDT interpretation was influenced by Gleason score and tracer used, and specifically highlighted pitfalls in rib and thoracic spine lesions, particularly on [18F]PSMA-1007 imaging [20]. These findings are consistent with the reduced performance of BUMP in our equivocal subgroup and support the continued importance of expert contextual assessment when interpreting borderline lesions.

There is an increasing drive towards standardised reporting within radiology. Multiple studies have identified that structured or proforma-based reporting significantly increases the completeness of cancer staging reports [21–25]. Initiatives driving standardised reporting are also being promoted for PSMA PET/CT reporting [26, 27]. A recent study examining the use of standardised scoring systems and frameworks in PSMA PET/CT reporting in patients referred to NYU Langone Health in the United States of America highlighted only 12% of reports (12 of 111) used a standardised reporting framework and in the context of nodal or distant metastases only 27% (*n* = 11) documented lesion size and 10% (*n* = 4) referred to corresponding CT characteristics [28]. This heterogeneity in reporting style could potentially lead to misinterpretation, diagnostic errors, inconsistent follow-up and management delays [24, 29].

The Standardised PSMA PET/CT Analysis and Reporting Consensus (SPARC) project aimed to combat heterogeneity in PSMA PET/CT through standardised reporting frameworks [27]. This included recommending the use of scoring systems such as PRIMARY score for intra-prostatic PSMA uptake patterns [30], PROMISE for standardised reporting of whole-body PSMA PET findings [31] and PSMA-RADS for structured reporting [8] among others. Our study adds to the growing evidence that PSMA-RADS v2.0 provides a clinically useful and reproducible framework for PSMA PET/CT interpretation. Recent validation studies have shown excellent reader agreement even among less experienced readers, supporting its broader implementation as a structured reporting tool [9, 32]. Nevertheless, persistent areas of difficulty within PSMA-RADS v2.0 indicate that standardisation should be viewed as a means of improving consistency rather than eliminating interpretative uncertainty.

An important contextual consideration is that most patients in this study cohort underwent PSMA PET/CT for post-treatment recurrence detection reflecting current commissioning policy from NHS England [33]. In this setting, UBU can often be managed more conservatively through watchful waiting or commencement of ADT. However, there is a strong evidence base for use of PSMA PET/CT for staging high-risk localised prostate cancer [7] and in this clinical scenario the implications of UBU differs. Therapeutic consequences of UBU in up-front staging are more immediate and potentially more significant for patient management [34]. Only a small proportion underwent imaging for initial staging, precluding a meaningful subgroup analysis by clinical indication. Consequently, the present findings primarily reflect the performance of BUMP and PSMA-RADS in the biochemical recurrence setting and should not be extrapolated uncritically to upfront staging. The integration of structured reporting to more holistically capture reporters’ certainty when describing osseous lesions could become even more important to mitigate overtreatment or delayed therapy in upfront staging. There is a paucity of evidence quantifying the impact of structured and standardised reporting systems on clinical decision making and patient outcomes related to PSMA PET/CT and this should be explored further.

The strengths of the study include its multicentre real-world design, with a reasonable cohort of bone lesions on PSMA PET-CT and moderate follow-up length. However, several limitations should be acknowledged. The retrospective design introduces inherent selection bias, the range of follow-up varied significantly with the shortest length of follow-up representing 40 days. Several lesions per patient were included and so the dataset likely contained intra-patient clustering. While patient level bootstrapping was used for ROC confidence intervals and performance thresholds, diagnostic performance metrics and confusion matrices were calculated at the lesion level without adjustment for this clustering and the precision of these estimates may therefore be mildly inflated. The study evaluated discrimination and threshold-based classification performance but did not formally assess calibration of BUMP as an absolute probability estimate. The reported BUMP values should therefore not be interpreted as calibrated lesion-level risks of malignancy in this cohort. The analysis of lesions labelled PSMA-RADS 3 was limited by a small sample size and the results should be interpreted cautiously. Generalisability is also limited to [18F]PSMA-1007, which has a higher prevalence of equivocal bone lesions but a lower malignancy rate than other PSMA radioligands; these findings should therefore not be directly extrapolated to other tracers [35]. A significant limitation in this area of research is in determining the ground truth of bone lesions. In this clinical scenario, histological proof of malignancy of bone lesions is rarely available and ground truth analysis relies on multidisciplinary consensus and assessment of the overall clinical picture through interrogation of the electronic patient record, which is often influenced by the reporter’s interpretation of PSMA PET/CT imaging findings. This increases the likelihood of incorporation bias. Six patients in this study were deemed to have lesions that were truly indeterminate and this represents a key patient group which were excluded from statistical analysis. Reader-specific experience was not recorded in sufficient detail to permit an experience-stratified analysis. The utility of BUMP scoring may be more prominent in less experienced reporters, and this should be explored.

## Conclusion

BUMP scoring performed well in predicting metastatic bone lesions but underperformed compared to expert structured PSMA-RADS v2.0 assessment. In equivocal lesions BUMP score offered limited value, highlighting the importance of expert interpretation for evaluating uncertainty raised by unspecific bone uptake.

## Supplementary Information

Below is the link to the electronic supplementary material.

Supplementary file1 (DOCX 1827 KB)

Supplementary file2 (XLSX 424 KB)

Supplementary file3 (DOCX 39 KB)

## Data Availability

The data that support the findings of this study are not openly available due to reasons of sensitivity.

## Declarations

Generative artificial intelligence tools were used solely to support data organisation and cross-referencing. No direct patient identifiers were entered, privacy-preserving configurations were used, and all outputs were independently verified against the source data by the authors.
