# Supplementary material for: Comparative analysis of PSMA-RADS Version 2.0 versus bone uptake metastatic probability (BUMP) score for characterising focal bone uptake on [18F]PSMA-1007 PET/CT in prostate cancer – a multicentre evaluation
Source: Eur J Nucl Med Mol Imaging. 2026 Sep 1;53(13):8448–62. doi: 10.1007/s00259-026-08154-0 (PMC13727886; doi:10.1007/s00259-026-08154-0)
Supplement: Supplementary file 1 [file 259_2026_8154_MOESM1_ESM.docx]

PSMA-PET/CT annotation protocol

# 1 Background

This work is part of the “End-to-end AI-assisted PSMA PET/CT reporting” InnovateUK Grant 10083748 which aims to develop tools to assist clinicians reporting PSMA PET/CT spanning disease detection, segmentation, characterization and structured reporting. It is a collaboration between Mirada Medical and 3 NHS Trusts..

# 2 Clinical annotation overview

The purpose of the clinical annotation work-package is to collect a variety of clinical information across a large number of PSMA PET/CT scans and subjects. In particular:

1. Clinical identification of individual image findings constituting the whole disease on the PSMA PET scans
2. Clinical characterization of individual image findings following a template based off published PSMA PET/CT reporting guidelines
3. Pre-PSMA PET clinical information
4. *Additional follow-up/outcome data**

**This will be collected in parallel/ in a second phase of the project.*

## 2.1 Input data

For each case, the input data consists of:

- At least one PSMA PET scan
- Associated CT scan
- Associated clinical report
- Associated pre-labeled disease RTSTRUCT file
- Associated reference regions RTSTRUCT file
- An excel file for capturing clinical information

Note: input data in black were provided by LTH. Input data in blue are additionally provided by Mirada for the clinical annotation work.

## 2.2 Tooling

### 2.2.1 Clinical labelling on PET/CT scans

The clinical labelling of the PSMA PET/CT scans will be performed using a research prototype version of XD which comes with the capability to load in the pre-labeled disease RTSTRUCT file which contains a list of structures generated as detailed in *Appendix 1*. On loading a PET, CT and the pre-labeled disease RTSTRUCT file containing the candidate findings, the structures populate a *Provisional Findings panel*.

### 2.2.2 Disease characterization

Disease characterization at the image finding and patient level will be captured using a Microsoft Excel spreadsheet per case.

## 2.3 Number of clinical readers

There should be a minimum of 2 readers per case. Reader 1 performs the clinical labelling on PET/CT scans, and disease characterization in the associated Excel file. Reader 2 reviews the work of Reader 1 and clinical readers reach consensus where needed.

Note: The Saved session capability in XD can be used by Reader 1 to save the list of clinically relevant image findings to be reviewed in the software application by Reader 2.

## 2.4 Output data

For each case, the output data will consists of:

1. A Final XD Saved session containing all individual findings constituting the whole disease on the PSMA PET scans
2. Associated Excel form filled in with full characterization of individual findings (*Individual image findings tab*) and additional clinical information at the patient level (*PatientForm tab*).
3. (Optionally) re-drawn reference regions in the XD Saved session.

# 3 Annotation Protocol

## 3.1 Loading data in the XD prototype

When loading a case in the XD prototype, also select the associated pre-labeled disease RTSTRUCT file (findings_BODYFX_SUV_4_7_10.dcm) and reference regions (reference_voi_BODYFX.dcm).

Note: If there are multiple PET reconstructions, use the conventional reconstruction rather than Q. Clear.

**Figure 1: Select PET, CT, pre-labelled disease and reference regions RTSTRUCT files**

## 3.2 Clinical markup of individual image findings

- All individual image findings should be segmented on the PET/CT scans.
- Each lesion should have a unique name in the XD *Findings* table.
- The same name should be used in the companion excel form (one entry per finding).

Any of the XD segmentation tools can be used to manually/semi-automatically segment PET or CT lesions. The set of pre-labelled hotspots provided as an RTSTRUCT file (findings_BODYFX_SUV_4_7_10.dcm) is intended as a starting point. It provides pre-segmented hotspots above an SUV of 4 following a red amber green (RAG) system and excluding findings in the liver, spleen, parotid glands and the small bowel, which can be used together with the XD PET hotspot user interface capabilities (see Appendix 1). Relevant provisional findings should be “accepted” using the XD PET hotspot UI capabilities to become regular Findings.

Notes:

- Findings with an SUV below 4, if deemed clinically important, will need to be added separately.
- Additional information about using the pre-labelled cases and/or PET hotspotter features are given in Appendix 1 and Appendix 3.

**Figure 2: Example of pre-labeled image findings on PET scan**

## 3.3 **Individual image findings characterization**

Each of the individual image findings (section 3.2) should have an associated entry (row) in the Excel file for that case under the *Individual image findings* tab, with matching Findings Name.

**Figure 3: Corresponding *Individual image findings* tab**

Note: Please complete every cell value from left to right using the dynamic drop-down menus and free text where appropriate.

## 3.4 Confirming SUVmean values in reference regions

The RSTRUCT file (reference_voi_BODYFX.dcm) contains reference regions (right parotid, descending aorta and spleen) to derive SUV_mean_ values for those regions (Appendix 2). We need you to confirm whether the regions are clinically suitable to be used as a reference (e.g. absence of lesions) and whether the automatic region was positioned appropriately or needs to be redrawn. Please document this in the *PatientForm* tab of the Excel file under the *Background reference Uptake* section.

**Figure 4: Background reference uptake section of the *PatientForm* tab**

Note: If the user(s) redraw one or more reference regions, those should be saved/exported from the XD prototype (see Section 3.5).

## 3.5 Exporting clinical image annotations

To export the image findings as an XD Saved Session file, navigate to File → Save Session…

Note: please give it a descriptive name.

**Figure 5: Exporting clinical image annotations as an XD Saved session**

# 4 Disease characterization at the patient level

Additional clinical information needs to be completed under the *PatientForm* tab of the spreadsheet. Open the patient annotation file and fill in the form in the first tab of the spreadsheet (see below). Readers must ensure that all relevant fields are filled in the patient form.

**Figure 6: Disease characterization and scan information - *PatientForm* tab**

# 5 Review by second radiologist

The second reader will need to review all clinical annotations performed by the first reader, and discuss to reach consensus where needed. Note: Any changes following Reader 2 review and reaching consensus, need to be reflected in the final XD saved session containing the image annotations, as well as the companion Excel file for that case.

# 6 Transfer of clinical annotations to Mirada

Once both readers have reviewed the case (and reached consensus where needed), the output data should be transferred to Mirada via secured FTP.

# 7 Appendix 1 - Using the pre-labelled findings with the PET hotspot functionality in the XD prototype

If the pre-labelled disease RTSTRUCT file (findings_BODYFX_SUV_4_7_10.dcm) is used, the findings will load in the ***Provisional Findings*** panel located in the bottom left corner in XD. Alternatively, if pre-labelled data is not used, the reader may generate provisional findings using the ***PET Hotspot Finder*** functionality of XD (see Appendix 3).

The reader can **accept** or **reject** provisional findings (individually or all). Once accepted, findings will be moved to the ***Findings*** panel. Subsequently, findings can be edited, and names can be updated as necessary.

Notes: Additional findings can be manually contoured by the reader if needed. Please refer to the Release Notes for additional detail about the PET Hotspot finder functionality.

## 7.1 How the pre-labels were generated

Provisional findings in the associated pre-labeled disease RTSTRUCT file (findings_BODYFX_SUV_4_7_10.dcm) were generated as followed:

1. A global fixed threshold of SUV of 4 was applied.
2. All findings smaller than 0.2mL were removed from the pool of candidates.
3. Hotspots that overlapped with organs of physiologic uptake (liver, spleen, kidneys, parotid glands and the small bowel) were discarded*
4. Remaining findings were color coded according to the level of PSMA uptake (Green: 4 < SUV_max_ ≤ 7, Amber: 7 < SUVmax ≤ 10, Red: SUVmax > 10).

Notes:

- The colour of the lesion contours are not automatically updated if the SUVmax falls in a different range of values following contour modifications.
- ** Hotspots that overlapped by more than 50% of their volume with at least one of the organs with high physiologic uptake were discarded. If lesions overlap with any of the beforementioned organs by less than 50%, a binary mask is applied, to remove the overlapping volume from the lesion mask. Lesions overlapping with the parotid glands are discarded, regardless of the overlap percentage.*

# 8 Appendix 2 - Automatic reference regions generation

Reference regions were automatically placed in the right parotid gland, descending aorta and in the spleen. Reference VOIs were generated as follows:

- Blood pool VOI is a 1.5cm diameter sphere, placed in the descending aorta 15 cm from the top
- Spleen VOI is a 2.5 cm diameter sphere placed in the spleen in the region with minimum coefficient of variation
- Parotid VOI is a 1.5 cm diameter sphere placed in the right parotid gland, in the region with minimum coefficient of variation

Reference regions are stored in a separate RTSSTRUCT file.

# 9 **Appendix 3: Generating a list of provisional findings with the** PET Hotspot Finder within XD (**fixed threshold)**

To generate provisional findings with a fixed threshold, click on the **PET Hotspot Finder** icon at the top right corner of the window. Note: the icon is active only when one of the PET views is selected.

In the PET Hotspot Finder window, fill in the desired value for an absolute threshold, and click OK.

This will populate the Provisional Findings panel with lesions having SUV values greater than the selected threshold.
