# Supplementary material for: Comparative analysis of PSMA-RADS Version 2.0 versus bone uptake metastatic probability (BUMP) score for characterising focal bone uptake on [18F]PSMA-1007 PET/CT in prostate cancer – a multicentre evaluation
Source: Eur J Nucl Med Mol Imaging. 2026 Sep 1;53(13):8448–62. doi: 10.1007/s00259-026-08154-0 (PMC13727886; doi:10.1007/s00259-026-08154-0)
Supplement: Supplementary file 2 [file 259_2026_8154_MOESM3_ESM.docx]

Supplemental Figure: Receiver operating characteristic curve demonstrating diagnostic performance of BUMP score in characterising equivocal PSMA-RADS 3 lesions.
